# Low‐normal birthweight in the first pregnancy as a risk factor for small for gestational age in the second pregnancy: A matched case–control study

**DOI:** 10.1002/pmf2.70335

**Published:** 2026-06-15

**Authors:** Boujenah Jeremy, Beddock Richard, Patrick Rozenberg

**Affiliations:** ^1^ Département d'obstétrique groupe hospitalier Diaconesses Croix Saint‐Simon Paris France; ^2^ Clinical Epidemiology Paris Saclay University UVSQ, Inserm, Team U1018, CESP Montigny‐le‐Bretonneux France; ^3^ Department of Obstetrics and Gynaecology American Hospital of Paris Paris France

**Keywords:** birthweight, recurrence risk: growth restriction, small for gestational age

## Abstract

**Background:**

Recurrent obstetric complications may stem from chronic placental dysfunction or maternal vulnerability, with potential worsening across successive pregnancies. Subtle intrauterine growth restriction in a first pregnancy, even within the appropriate‐for‐gestational‐age (AGA) range, may signal underlying risk for subsequent adverse outcomes.

**Objectives:**

To determine whether low‐normal birthweight in a first AGA pregnancy is associated with increased risk of small‐for‐gestational‐age (SGA) birth in the second pregnancy among women with two consecutive term singleton births.

**Methods:**

We conducted a retrospective matched case–control study nested within a historical cohort of all births between 2014 and 2024. All cases (*n* = 125) that delivered a first AGA and a second SGA infant were included. Controls (*n* = 250) who delivered two consecutive AGA infants, matched 1:2 on infant sex and date of birth of the second child. Primary exposure was standardized birthweight *Z*‐score of the first child, categorized as <−0.5 standard deviation (SD) or <−1 SD. Primary outcome was SGA birth (<10th percentile) in the second pregnancy. Logistic regression, adjusted for maternal age, prepregnancy weight, height, ethnicity, smoking, socioeconomic status, and time to conception, and generalized structural equation model (GSEM) accounted for within‐woman correlation were performed.

**Results:**

Among 375 women analyzed, first‐born infants of cases had lower mean birthweight than controls (3072 vs. 3375 g). Compared with reference birthweight (*Z*‐score ≥−0.5 SD), first‐born *Z*‐score <−0.5 SD was associated with fivefold increased odds of subsequent SGA (adjusted odds ratio [aOR], 5.11; 95% confidence interval [CI], 2.51–10.41). Risk increased for *Z*‐score <−1 SD (aOR, 8.52; 95% CI, 3.40–21.30), demonstrating dose–response (OR for trend 3.32; 95% CI, 2.16–5.10). GSEM confirmed findings (aOR, 5.46 and 8.00, respectively; *ρ* = 0.33). Quantitative bias analysis suggested that unmeasured confounding was unlikely to explain the observed associations.

**Conclusions:**

Low‐normal birthweight in a first AGA pregnancy is associated with subsequent SGA in the subsequent pregnancy suggesting an undetected placental or maternal vulnerability.

## 1 INTRODUCTION

Fetal growth follows a continuum, and the conventional threshold of the 10th birthweight (BW) percentile used to define small for gestational age (SGA) is inherently arbitrary [1]. Recent evidence suggests that adverse perinatal outcomes extend below the 25th–30th percentile, questioning whether infants born at the lower end of the normal range, the so‐called appropriate for gestational age (AGA), are truly at low risk [2].

The relationship between parity and BW has been shown to be nonlinear with an important change observed between first‐ and second‐born infants of the same mother, persistent after adjustment over interpregnancy maternal characteristics [3]. As a result, a multiparous woman with a prior AGA birth is typically classified as low risk for SGA in subsequent pregnancies [4].

Most of the studies focused on the risk of recurrent obstetrical adverse outcomes, such as diabetes, hypertensive disorder [5], preterm delivery [6], or on the relation between such complication in the first pregnancy and the risk of a different one in the second [7]. A dose relationship between the first BW percentile and the risk of subsequent preeclampsia [8] and SGA has been observed [6].

Recurrent obstetric complications may stem from a common etiologic basis such as chronic placental dysfunction or maternal vulnerability, which enhance adverse outcomes across pregnancies. Therefore, a risk pattern for SGA may sometimes be revealed as pregnancies are repeated. Some authors found that a short interval interpregnancy [9], changing partner [10], black ethnicity [11], and an interpregnancy body mass index (BMI) decrease [12] increase the risk of subsequent SGA. Hence, giving birth to an SGA child after a first uncomplicated pregnancy is an unexpected occurrence and remains a challenge for diagnosis and prediction

To clarify whether a low‐normal BW in the first pregnancy is associated with an increased risk of SGA in a subsequent pregnancy, we designed a matched case‐control study.

## 2 MATERIAL AND METHODS

### 2.1 Source of the population

We conducted a case–control study nested within a historical cohort of all births occurring between January 2014 and May 2024. The beginning of the study was chosen according to the available medical chart that are stored in the archive records. The end of the study was chosen according to the beginning of conceptualization of the study. All data were carried out in a French maternity unit of level 1 (facility dedicated to low‐risk pregnancies and normal deliveries, providing obstetric monitoring and routine neonatal care without a neonatal intensive care unit). The data of the maternity unit are broadly representative of the low‐risk French population in terms of BW, maternal age, term delivery, and rate of late SGA.

SGA was defined as a BW below the 10th sex‐specific percentile at birth, according to the French AUDIPOG (Association des Utilisateurs de Dossiers Informatisés en Pédiatrie, Obstétrique et Gynécologie) reference curves.

All included cases and controls had research‐acceptable data, defined by an institutional quality control process that identifies and excludes patients with non‐continuous follow‐up or poor data recording raising suspicion as to the validity of that patient's record. All variables were manually extracted from individual medical records by a single investigator, ensuring exhaustive and consistent data collection. As required by French law and regulations, the study and the database were approved by the National Data Protection Authority (Commission Nationale de l'Informatique et des Libertés—CNIL). Because the dataset contained no data enabling patient identification and all women received standard care, the study was exempt from informed consent requirements.

### 2.2 Study population and selection of patients

To be eligible, women were required to have two consecutive singleton live births after 36 weeks of gestation in our maternity unit, with a first BW above the 10th sex‐specific percentile (i.e., non‐SGA), according to French AUDIPOG reference curves. Among women meeting the above inclusion criteria (i.e., first child AGA), cases and controls were selected as follows.

Cases were defined as follows: multiparous women who had the first two consecutive childbirths (number 1 and 2) in the same maternity unit with a first AGA and a second SGA childbirths (defined as BW below the 10th percentile according to the AUDIPOG reference curves) (AGA/SGA). To avoid selection bias with a low risk of differential selection based on exposure, all eligible cases were included.

Controls were randomly selected by pairing the case (1:2) and were defined as follows: a multiparous women who had the first two consecutive childbirths (number 1 and 2) in the same maternity unit with a first and a second AGA childbirths (AGA/AGA) and matched on sex and date of birth (± 1 day if necessary for feasibility) of the second child. Matching on sex and birth date of the second child helped control for temporal and sex‐adjusted BW precision. The selection of controls was conducted independently of the exposure (the first BW), ensuring an unbiased estimate of the association. In addition, a propensity score, based on sex and BW of both children, interpregnancy interval, and maternal age was applied to assess the balance between participating and non‐participating control women to minimize potential bias in the random selection of controls.

Exclusion criteria were as follows: first delivery in another maternity unit, twin pregnancies, stillbirth at the first delivery, first delivery with SGA and major congenital anomalies in one of the pregnancies, women with SGA childbirth at the first and second pregnancies (SGA/SGA), and women with SGA and AGA childbirths at the first and second pregnancies (SGA/AGA).

As all eligible cases were exhaustively included, no a priori sample size calculation was performed, consistent with standard practice for exhaustive case–control designs.

### 2.3 Data collection

To compare case and control, the following variables of interest were collected for each pregnancy:

− paternal variables: age, chronic disease, ethnic origin, height, change of male partner
− maternal variables: age, weight at the conception, height, weight gain, interpregnancy BMI change, method of conception (spontaneous or after medically assisted reproduction), duration for conception, smoking status before pregnancy and during pregnancy, previous and number of miscarriages, previous chronic maternal disease, hypothyroidism, endometriosis disease, previous uterine surgery, blood group and D rhesus status, special diet (self‐reported vegan or vegetarian dietary restrictions), intravenous iron treatment, psychologic disorder, economic status defined by employment status and eligibility for universal health coverage, breastfeeding, systolic and diastolic blood pressure in the first trimester.
− Obstetrical and neonatal variables: interpregnancy interval, cytomegalovirus status, PAPP‐A or free β‐hCG levels between 11+ 0 and 13 + 6 weeks (mom), estimated percentile of fetal weight at second and third trimesters, notch at uterine doppler (second trimester) fetal pathology (minor fetal conditions not known to affect fetal growth), gestational age at birth, type of onset labor (spontaneous or induce), mode of delivery, etiology for cesarean delivery, type of presentation at birth (breech or cephalic), BW sex adjusted, mode of delivery, neonatal pathology, pH, and lactate value at birth

For the estimated fetal weight at ultrasound diagnosis, the national French curve of CFEF was used to determine the percentile of growth [13]. At birth, the French sex‐specific AUDIPOG charts accessible online were used in maternity unit to evaluate BW for term infants [14, 15].

### 2.4 Hypothesis and exposure variable

Our hypothesis was among sibling pairs where the first child was AGA, having a “low‐normal BW” for the first child is associated with an SGA in the second child. Thus, this could reflect a subtle non‐physiologic deviation from expected fetal growth in the first pregnancy which would be more pronounced during subsequent pregnancy.

The primary outcome was the presence of SGA in the second child, and the main exposure was a low‐normal first BW.

BW was converted into a sex‐ and gestational‐age‐specific *Z*‐score using French AUDIPOG national reference curves. Since LMS parameters are not publicly available in digital format, *Z*‐scores were derived by interpolation of the published charts assuming a normal distribution. Two clinically meaningful thresholds were used to define low‐normal BW: −0.5 standard deviation (SD) (approximately the 31st percentile) and −1.0 SD (approximately the 16th percentile)

These thresholds were used as the primary binary exposure for the analysis. This categorization was used to allow assessment of a potential dose–response relationship between increasing exposure severity and the outcome.

### 2.5 Statistical analysis

Descriptive statistics included means and SD, or medians and interquartile ranges (IQRs) for continuous variables, and frequencies with percentages for categorical variables. Categorical variables were compared using Fisher's exact test and continuous variables using the Welch test or Wilcoxon rank‐sum test as appropriate. Odds ratios (ORs) and 95% confidence intervals (CIs) were estimated for associations of interest.

#### 2.5.1 Primary analysis

We first compared the mean BW of the first child between cases and controls. We then compared the classification performance of the two *Z*‐score thresholds (<−0.5 SD and <−1.0 SD) using McNemar's test.

To examine whether low‐normal first BW was an independent risk factor for SGA in the second pregnancy, unconditional multivariable logistic regression was used. Although cases and controls were matched 1:2 on infant sex and delivery date of the second child, conditional logistic regression was not used. These matching variables are characteristics of the outcome pregnancy (temporally downstream of the exposure) and cannot be confounders in our causal framework based on the directed acyclic graph (DAG). Moreover, conditioning on delivery date would have generated many unique strata, leading to sparse data bias [16]. Confounders were selected using DAGs: maternal age, height, prepregnancy weight, smoking, ethnicity, time to conception (TTC), and socioeconomic status were included [17, 18, 19]. Only the OR for the primary exposure was interpreted to avoid the “Table 2 fallacy” [16].

To account for the non‐independence of observations across two pregnancies, a generalized structural equation model (GSEM) was used to jointly estimate the association between the categorical exposure and both outcomes while explicitly modeling the correlation of unmeasured residuals (*ρ*), which may capture shared latent factors such as uteroplacental insufficiency.

#### 2.5.2 Effect modification

Paternal age and height, maternal blood group, family history of hypertensive disorders, interpregnancy interval (IPI), and interpregnancy BMI change were explored as potential effect modifiers based on prior literature [20, 21]. Paternal age was dichotomized at 40 years and paternal height at 170 cm (approximating the 25th percentile of the French male population). IPI was categorized as short (< 12 months), reference (12–48 months), and long (> 48 months) [22]. Interpregnancy BMI change was treated as a continuous variable and analyzed using marginal effects to detect potential non‐linear patterns.

#### 2.5.3 Sensitivity analyses

To test the robustness of exposure and outcome definitions, SGA and *Z*‐scores were recalculated using the Fenton and INTERGROWTH‐21st growth standards, and associations were re‐estimated using the same adjusted logistic regression model.

To account for within‐woman correlation across pregnancies, two additional models were fitted: a logistic regression with patient‐level cluster‐robust standard errors and a random‐intercept mixed‐effects logistic regression model to estimate the intraclass correlation coefficient (ICC) and quantify the proportion of outcome variance attributable to unmeasured between‐woman factors.

### 2.6 Bias analysis

To assess the potential impact of unmeasured confounding, the E‐value was calculated for the observed OR and its lower confidence bound [23]. We subsequently conducted a quantitative bias analysis (QBA) including a Monte Carlo simulation, specifying plausible distributions for the prevalence and effect of potential unmeasured confounders among exposed and unexposed individuals.

## 3 RESULTS

### 3.1 Study population

**FIGURE 1:**
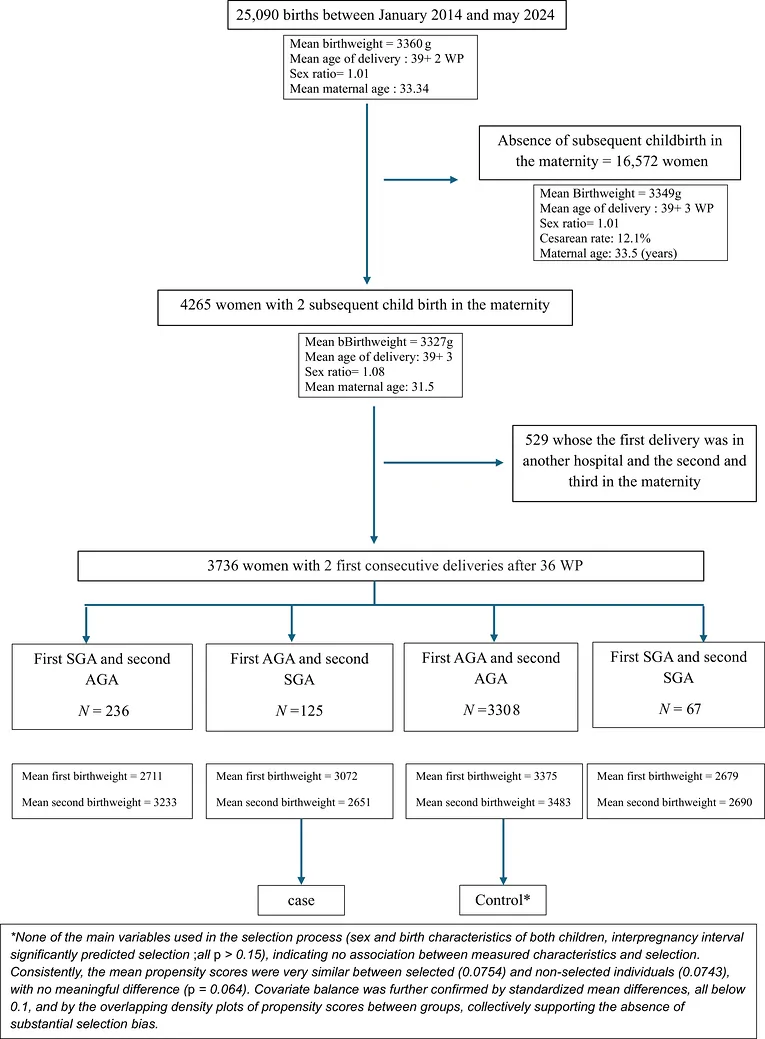
Flow chart. AGA, appropriate for gestational age; SGA, small for gestational age; WP, weeks of pregnancy.

Between 2014 and 2024, 25,090 births occurred, of whom 3736 women were eligible for the study. After excluding women with two consecutive SGA births (*n*  =  67) and women with a first SGA birth followed by an AGA birth (*n*  =  236), 3433 women remained eligible for case and control selection. A total of 125 cases (AGA/SGA) and 250 controls (AGA/AGA) were included in the final analysis (Figure 1).

### 3.2 First pregnancy BW

The mean BW of the first child was significantly lower in cases than in controls (3072 vs. 3375 g, *p* < 0.05)

### 3.3 Characteristics of cases and controls

Univariate analysis comparing cases and controls at the first pregnancy identified several clinically and statistical meaningful differences. Cesarean delivery was more frequent in cases (21.2% vs. 12.4%), particularly for abnormal fetal heart rate tracing (77% vs. 22.6%). Cases also had a higher rate of hypertensive disorders (3.2% vs. 0%) and uterine artery notching at the second trimester Doppler (8.8% vs. 2.4%) (Tables 1 and 2).

**TABLE 1:** Characteristics of the first pregnancy.

	Case *n* = 125 Median (IQR) or *n* (%)	Control *n* = 250	*p*
**First pregnancy birthweight**			
Birthweight			
Weight (g)	3075 (2865–3245)	3362 (3140–3621)	0.01
Percentile	26 (18–42)	54 (33–72)	
*Z*‐score > −0.5 SD	40 (36.0%)	192 (76.8%)	
*Z*‐score −0.5 to −1.0 SD	49 (39.2%)	43 (17.2%)	
*Z*‐score < −1.0 SD	31 (24.8%)	15 (6.0%)	
**Paternal characteristics**			
Age			
Median age (years)	33 (30–36)	32.5 (30–35.25)	0.37
≥40 years old	10 (8.8%)	22 (8.8%)	0.87
Ethnicity			
Non‐Caucasian	20 (16.0%)	39 (15.6%)	0.88
Height			
Median height (cm)	177 (172–181)	180 (175–185)	0.01
≤170 cm	21 (16.8%)	27 (10.8%)	0.07
**Maternal characteristics**			
Age			
Median age (years)	32 (30–34)	31 (30–34)	0.68
Height			
Median height (cm)	164 (160–168)	166 (163–168)	0.01
Weight			
Prepregnancy weight (kg)	55 (51–62)	60 (54–65)	0.01
Loss of pregnancy			
Previous miscarriage	30 (24.3%)	60 (24.0%)	1.00
≥2 miscarriages	5 (16.6%)	6 (10.0%)	0.49
Before first pregnancy	16 (53.3%)	19 (31.6%)	0.02
After first pregnancy	14 (46.6%)	49 (68.3%)	
Ectopic pregnancy	3 (2.4%)	3 (1.2%)	0.40
Conception			
After ART	14 (11.2%)	16 (6.4%)	0.11
Delay to conceive (months)	5 (3–9)	4 (2–7)	0.12
Smoking			
Before/between pregnancies	34 (27.2%)	47 (18.8%)	0.08
During pregnancy	11 (8.8%)	8 (3.2%)	0.02
Socioeconomic			
Low socioeconomic status	37 (29.6%)	27 (10.8%)	0.01
Medications			
L‐thyroxin	4 (3.2%)	1 (0.4%)	0.04
Aspirin	1 (0.8%)	2 (0.8%)	1.00
Antidepressant	1 (0.8%)	1 (0.4%)	1.00
Medical history			
Family history of hypertensive disorder	29 (23.2%)	38 (15.2%)	0.06
Chronic disease (asthma, migraine)	2 (1.6%)	9 (3.6%)	0.75
Endometriosis	3 (2.4%)	8 (3.2%)	0.75
Hypothyroidism	6 (4.8%)	13 (5.2%)	1.00
Previous uterine/cervical surgery	14 (11.2%)	22 (8.8%)	0.46
Blood group			
Non‐O	75 (60.0%)	144 (57.6%)	0.73
O	50 (40.0%)	106 (42.4%)	
Rhesus D			
Positive	112 (89.6%)	214 (85.6%)	0.33
Negative	13 (10.4%)	36 (14.4%)	
**Obstetrical characteristics**			
Weight gain			
Weight gain (kg)	11 (8–14)	13 (10–16)	0.01
First trimester markers			
HCG 11–13 WP (MoM)	1.06 (0.66–1.7)	1.1 (0.75–1.7)	0.44
PAPP‐A 11–13 WP (MoM)	1.0 (0.71–1.39)	1.07 (0.75–1.5)	0.08
CMV			
Previous immunization	64 (51.2%)	109 (43.6%)	0.18
Ultrasound			
Percentile at 2nd ultrasound	50 (35–60)	51.5 (36–70)	0.13
Percentile at 3rd ultrasound	35 (21–59)	52.5 (33–73)	0.01
Deceleration > 1 quartile (2nd→3rd US)	24 (19.2%)	37 (14.8%)	0.30
Deceleration > 2 quartiles (2nd→3rd US)	4 (3.2%)	3 (1.2%)	0.22
Uterine Doppler			
Notch at uterine Doppler	7 (5.6%)	11 (4.4%)	0.61
Blood pressure			
Highest systolic BP at 15 WP (mmHg)	111 (108–120)	112 (108–121)	0.52
Highest diastolic BP at 15 WP (mmHg)	66 (60–70)	67 (60–71)	0.61
Complications			
Any pregnancy complication	15 (13.7%)	19 (7.6%)	0.18
Gestational diabetes	9 (60.0%)	14 (73.7%)	0.58
Hypertensive disorder	4 (26.7%)	2 (10.5%)	
Threatened preterm	2 (6.6%)	1 (5.3%)	
Thombopenia	2 (6.6%)	2 (10.5%)	
**Labor, intrapartum, and neonatal characteristics**			
Gestational age			
At delivery (days)	273 (266–280)	280 (273–280)	0.08
Labor			
Induced labor	18 (14.4%)	44 (17.6%)	0.46
Indication: prolonged rupture of membranes	7 (38.8%)	18 (40.9%)	0.02
Indication: prolonged pregnancy	5 (27.8%)	23 (52.2%)	
Indication: maternal/fetal pathology	6 (32.4%)	3 (6.9%)	
Mode of delivery			
Vaginal delivery	99 (79.2%)	219 (87.2%)	0.04
Cesarean section	26 (21.2%)	31 (12.4%)	
CD indication			
Abnormal fetal heart rate tracing	20 (77.0%)	7 (22.6%)	0.01
Arrest of labor	4 (15.0%)	16 (51.6%)	
Breech presentation	2 (8.0%)	8 (25.8%)	
Neonatal			
Acidosis at birth (pH < 7.05 and/or lactates >10)	8 (6.4%)	9 (3.6%)	0.29
Breastfeeding	99 (79.2%)	195 (78.0%)	0.89
Sex			
Male	73 (58.4%)	127 (50.8%)	0.18
Female	52 (41.6%)	123 (49.2%)	

*Note*: Data presented as median (IQR) for continuous variables and *n* (%) for categorical variables. *p* values are from Fisher's exact test or Wilcoxon rank‐sum test, as appropriate.

Abbreviations: ART, assisted reproductive technology; BP, blood pressure; CMV, cytomegalovirus; CS, cesarean section; IQR, interquartile range; MoM, multiple of the median; SD, standard deviation; US, ultrasound; WP, weeks of pregnancy.

**TABLE 2:** Characteristics of the second pregnancy.

	Case *n* = 125 Median (IQR) or *n* (%)	Control *n* = 250	*p*
**Second pregnancy birthweight**			
Birthweight			
Weight (g)	2730 (2572–2850)	3497 (3190–3766)	0.01
Percentile	5 (3–8)	62 (37–80)	0.01
**Interpregnancy interval**			
IPI			
Median IPI (months)	23.5 (15–35)	22 (14–31)	0.04
Optimal (18–48 months)	78 (62.4%)	139 (55.6%)	0.08
Short (< 18 months)	36 (28.8%)	98 (39.2%)	
Long (> 48 months)	11 (8.8%)	11 (4.4%)	
**Paternal characteristics**			
Age			
Median age (years)	36 (33–39)	36 (33–39)	0.61
≥40 years old	31 (24.8%)	49 (19.6%)	0.25
Partner			
Change of male partner	3 (2.4%)	0 (0%)	0.03
Maternal characteristics			
Age			
Median age (years)	34 (32–37)	33.5 (31.2–36.2)	0.21
≥35 years old	48 (38.4%)	91 (36.4%)	0.73
Conception			
After ART	12 (9.6%)	10 (4.0%)	0.03
ART after first spontaneous pregnancy	4 (3.2%)	4 (1.6%)	0.44
Time to conceive (months)	6.4 (—)	4.5 (2–6)	0.01
Weight			
Prepregnancy weight (kg)	56 (51–61)	60 (54–68)	0.01
Interpregnancy weight change			
Stable (−2 to +5 kg)	96 (76.8%)	176 (70.4%)	0.14
Weight loss < −2 kg	20 (16.0%)	39 (15.6%)	
Weight gain > +5 kg	9 (7.2%)	35 (14.0%)	
Smoking			
During pregnancy	8 (6.4%)	7 (2.8%)	0.10
Medications			
L‐thyroxin	1 (0.8%)	3 (1.2%)	1.00
Aspirin	2 (1.6%)	1 (0.4%)	0.25
Antidepressant	1 (0.8%)	0 (0%)	0.33
Medical history			
Hypothyroidism	3 (2.4%)	12 (4.8%)	0.40
Psychological care	10 (8.0%)	13 (5.2%)	0.36
**Obstetrical characteristics**			
Weight gain			
Weight gain (kg)	10 (7.5–12.5)	12 (10–15)	0.01
First trimester markers			
HCG 11–13 WP (MoM)	1.31 (0.76–2.1)	1.09 (0.71–1.64)	0.03
PAPP‐A 11–13 WP (MoM)	0.97 (0.63–1.4)	1.06 (0.74–1.5)	0.03
CMV			
Previous immunization	74 (59.2%)	129 (51.6%)	0.58
Interpregnancy CMV immunization	12 (9.6%)	28 (11.2%)	0.72
Ultrasound			
Percentile at 2nd ultrasound	36 (24–50)	52 (37–73)	0.01
Percentile at 3rd ultrasound	20 (12–35)	57.5 (38–79)	0.01
Deceleration > 1 quartile (2nd→3rd US)	36 (28.8%)	30 (12.0%)	0.01
Deceleration > 2 quartiles (2nd→3rd US)	5 (4.0%)	1 (0.4%)	0.01
Uterine Doppler			
Notch at uterine Doppler	11 (8.8%)	6 (2.4%)	0.01
Blood pressure			
Highest systolic BP at 15 WP (mmHg)	112 (108–123)	112 (105–120)	0.34
Highest diastolic BP at 15 WP (mmHg)	66 (59–72)	64 (58–70)	0.10
Complications			
Gestational diabetes	12 (9.6%)	16 (6.4%)	0.29
Hypertensive disorder	4 (3.2%)	0 (0%)	0.01
Threatened preterm	0 (0%)	3 (1.2%)	0.55
**Labor, intrapartum, and neonatal characteristics**			
Gestational age			
At delivery (weeks)	39 (39–40)	39 (39–40)	0.97
Labor			
Induced labor	23 (16.8%)	27 (10.8%)	0.01
Indication: prolonged rupture of membranes	0 (0%)	6 (2.4%)	0.01
Indication: prolonged pregnancy	2 (1.6%)	15 (6.0%)	
Indication: hypertensive, GDM or SGA	21 (16.8%)	6 (2.4%)	
Mode of delivery			
Vaginal delivery	119 (95.0%)	220 (88.0%)	0.02
Cesarean section	6 (4.8%)	30 (12.0%)	
CD indication			
Abnormal fetal heart rate tracing	3 (50.0%)	4 (13.3%)	0.18
Arrest of labor	0 (0%)	5 (33.4%)	
Systematic (breech or previous CS)	3 (50.0%)	16 (53.3%)	
Neonatal			
Acidosis at birth (pH < 7.05 and/or lactates >10)	5 (4.0%)	5 (2.0%)	0.31
Sex			
Male	60 (48.0%)	120 (48.0%)	1.00
Female	65 (52.0%)	130 (52.0%)	
Sex concordance			
Same sex in both pregnancies	68 (54.4%)	124 (49.6%)	0.44
Boys then boys	39 (57.3%)	59 (47.5%)	0.01
Girls then girls	29 (42.7%)	65 (52.5%)	
Boy then girl	34 (27.2%)	68 (27.2%)	1.00
Girl then boy	22 (17.6%)	58 (23.2%)	0.07

*Note*: Data presented as median (IQR) for continuous variables and *n* (%) for categorical variables. *p* values are from Fisher's exact test or Wilcoxon rank‐sum test, as appropriate.

Abbreviations: ART, assisted reproductive technology; BP, blood pressure; CMV, cytomegalovirus; CS, cesarean section; GDM, gestational diabetes mellitus; IPI, interpregnancy interval; IQR, interquartile range; MoM, multiple of the median; SGA, small for gestational age; US, ultrasound; WP, weeks of pregnancy.

### 3.4 Comparison of *Z*‐score thresholds

The two exposure thresholds classified significantly different proportions of children (χ^2^(1) = 90.0, *p* < 0.001). All children identified by the stricter threshold (*Z* < −1.0 SD) were also captured by the −0.5 SD threshold. The less stringent criterion (*Z* < −0.5 SD) additionally identified 92 children (24.5% of the cohort) not classified under the −1.0 SD cutoff.

In crude analyses, both thresholds were associated with an increased risk of SGA in the second pregnancy: OR, 5.88 [95% CI, 3.68–9.40] for *Z* < −0.5 SD and OR, 5.16 [95% CI, 2.66–10.00] for *Z* < −1.0 SD.

### 3.5 Multivariable analysis

In multivariable logistic regression, a dose–response relationship was observed between increasing exposure severity and the risk of subsequent SGA. Compared with women whose first child had a BW ≥−0.5 SD, adjusted odds ratios (aORs) were 5.11 (95% CI, 2.51–10.41) for the −0.5 to −1.0 SD group and 8.52 (95% CI [3.40–21.30]) for the >−1.0 SD group, with evidence of a significant linear trend (aOR for trend = 3.32; 95% CI, 2.16–5.10]).

Results from the GSEM were consistent with the main analysis: aORs were 5.46 (95% CI, 2.57–11.6) for the −0.5 to −1.0 SD group and 8.00 (95% CI, 3.71–17.20]) for the > −1.0 SD group. The correlation of unmeasured residuals was moderate (*ρ* = 0.33; 95% CI, 0.23–0.41), suggesting a shared latent biological factor across pregnancies (Table 3).

**TABLE 3:** Association between first pregnancy birthweight *Z*‐score and risk of SGA in the second pregnancy.

Exposure level (birthweight *Z*‐score)	Crude OR (95% CI)	Adjusted OR^a^ (95% CI) + linear trend test^b^	GSEM model^c^ aOR (95% CI)+
>−0.5 SD (Reference)	Reference	Reference	Reference
−0.5 to −1.0 SD	4.86 (2.88–8.20)	5.11 (2.51–10.41)	5.46 (2.57–11.60)
<−1.0 SD	8.82 (4.39–17.70)	8.52 (3.40–21.30)	8.00 (3.71–17.20)
Linear trend^b^	—	aOR = 3.32 (95% CI, 2.16–5.10)	—
Correlation of unmeasured residuals (ρ)	—	—	0.33 (95% CI, 0.23–0.41)

Model fit: Pseudo *R* ^2^ = 0.43; AUROC = 0.90; Hosmer–Lemeshow test, *p* = 0.58; Likelihood ratio test, *p* < 0.001.

Abbreviations: aOR, adjusted odds ratio; AUROC, area under the receiver operating characteristic curve; CI, confidence interval; GSEM, generalized structural equation model; OR, odds ratio; SD, standard deviation; SGA, small for gestational age.

^a^ Multivariable logistic regression adjusted for maternal age, maternal height, prepregnancy weight, smoking during pregnancy, ethnicity, time to conceive, and socioeconomic status.

^b^ Linear trend test using exposure level as an ordinal continuous variable in the adjusted model.

^c^ GSEM jointly estimating the association between categorical birthweight exposure and subsequent SGA while modeling the correlation of unmeasured residuals (*ρ*) as a proxy for shared latent factors (e.g., placental dysfunction) across pregnancies.

### 3.6 Effect modification

A shorter IPI (<12 months) and a longer IPI (>48 months) were both associated with a higher risk of subsequent SGA among women exposed to the −0.5 SD threshold (aOR, 37.9; 95% CI, 5.79–386.6) and 8.89 (95% CI, 1.07–83.83), respectively). A test for interaction suggested effect modification by exposure status (*p* = 0.05). Marginal effects analysis indicated that the impact of prior low BW declined with increasing maternal BMI (*p* < 0.05), with the strongest effect observed at low BMI (Table S1 and Figure S2).

### 3.7 Sensitivity analyses

Sensitivity analyses using alternative growth standards (Fenton and INTERGROWTH‐21st) showed consistent dose‐response associations (Table S2). Alternative modeling approaches confirmed the main GSEM findings. The mixed‐effects model showed an intraclass correlation coefficient (ICC) close to zero, indicating negligible between‐woman variability and supporting a fixed underlying mechanism rather than random maternal heterogeneity (Table S3).

### 3.8 Bias analysis

In the fully adjusted model, the E‐value for the observed OR (5.11) was 9.7, with a lower bound of 4.5 for the 95% CI. These findings indicate that an unmeasured confounder would need to be associated with both the exposure and the outcome by a risk ratio of at least 4.5 to fully explain the observed association. Quantitative bias analysis including Monte Carlo simulation confirmed the robustness of the findings across a wide range of scenarios (Table S4).

## 4 DISCUSSION

### 4.1 Main results

In this matched case–control study, a low‐normal BW in the first pregnancy was associated with increased risk of SGA in the second, despite initially AGA status. This dose–response relationship persisted after adjustment for measured confounders and remained consistent in GSEM, which accounted for within‐women dependencies and unmeasured latent factors, consistent with the hypothesis of a shared latent biological factor across pregnancies, most plausibly related to uteroplacental function

### 4.2 Interpretation

The correlation of residuals in the GSEM indicates that low BW and SGA share unexplained variance after adjustment for measured covariates, suggesting the influence of a shared latent biological factor not captured in the model, which may reflect underlying uteroplacental dysfunction. The higher rate of the cesarean delivery for non‐reassuring fetal heart rate in the first pregnancy and the increased occurrence of hypertensive disorder in the second pregnancy underline our hypothesis.

Our findings are consistent with those of Lykke et al., who reported a similar dose–response relationship between first pregnancy BW and subsequent SGA (OR, 2.40 for −0.5 to −1.0 SD and OR, 4.88 for ≥−1.0 to −1.5 SD), supporting the biological plausibility of our results in an independent cohort [6].

These results reinforce the concept of a fetal growth continuum. Population‐based studies [24, 25] have shown that the lowest perinatal morbidity occurs between the 50th and 84th percentiles, and that neonatal morbidity increases below the 25th percentile, well above the conventional 10th percentile SGA threshold [26]. From a histological standpoint, maternal vascular malperfusion lesions have been shown to correlate inversely with BW even in AGA neonates [27], and such lesions are known to recur in 10%–25% of cases, providing a plausible biological substrate for the recurrence pattern observed in our study [28]. Overall, our hypothesis is consistent with published histological and clinical evidence but not directly demonstrated by our data.

### 4.3 Clinical and research perspective: A low eutrophic first BW as a marker of incident SGA in the second pregnancy

All newborn with 10th–30th percentile cannot be considered as at risk because of the large number of women. Therefore, it is crucial to identify women with a prior infant below −0.5 SD at risk of subsequent SGA, enabling more tailored antenatal monitoring and interventions in subsequent pregnancies, potentially improving perinatal outcomes. As such and in line with our observation, women with a low first BW child born with cesarean delivery for non‐reassuring fetal heart rate tracing represent a subgroup that may warrant closer antenatal surveillance in a subsequent pregnancy. However, prospective studies are needed before any formal screening strategy can be recommended. As several and interacting conditions may alter fetal growth, women with low BMI could be advised to avoid a short IPI and to have a global evaluation of their nutrition.

Some baby could be born at the right time but not at the right size. This reinforces the concept of a fetal growth continuum and supports that some fetus may have late growth deceleration, but not severe or rapid enough to be recognized and low BW. The question raised with the concept of hidden eutrophic IUGR is: How to identify before or at birth these infants?

From an ultrasound perspective, instead of arbitrary/conventional threshold and alongside the classical definition of IUGR, an expected percentile trajectory with uterine doppler and late cerebroplacental doppler could be advised and detect more fetus at risk than an estimated fetal weight alone. At birth, a focus on the ratio of BW to placental weight (BW:PW) could be useful, as it has been described as a proxy of uteroplacental efficiency [29] and related to several adverse outcome [30].

Future studies should consider using a broader definition of adverse fetal growth in the second pregnancy, incorporating antenatal growth deceleration and Doppler criteria alongside BW‐based SGA, as this may capture a larger proportion of fetuses affected by recurrent uteroplacental dysfunction.

### 4.4 Strength and limitations

Strengths of the study include its design nested within a real‐life, low‐risk maternity setting, capturing all eligible deliveries after 36 weeks without selection based on pathology. This population‐based approach minimizes selection and participation biases, notably those linked to preterm delivery or referral. The matched case–control design (both on sex and date of birth), the multivariable modeling with DAGs, enhanced the robustness and causal interpretability of the findings and preserved the internal validity of the study. Hence, the difference in the first BW confirmed the expected results and supported the validity of case and control selection.

Quantitative bias analysis suggests that no plausible unmeasured confounder alone (as partner change between pregnancies, non‐cytomegalovirus (CMV) infections, alcohol or toxic exposures, special diet) is likely to fully explain the observed associations. Assisted reproductive technology (ART) was measured and could be a confounding factor but we deliberately chose not to exclude ART pregnancies. We accounted for subfertility by including TTC as a covariate in our multivariable model, which serves as a proxy for subfertility and ART use, and we examined ART use in the second pregnancy as a descriptive variable and tested for effect modification. Although cases more frequently conceived their first pregnancy via ART, there was no excess ART use for the second pregnancy among cases compared to controls.

The study period was determined by data availability at both ends, not by clinical or methodological considerations. However, temporal heterogeneity does not represent a meaningful source of bias in our study for the following reasons:

French national guidelines for the definition and management of SGA were published in 2013 (CNGOF recommendations), prior to the start of our study period, and remained unchanged throughout. The overall rate of SGA in our maternity unit remained stable across the study period, arguing against any meaningful change in case ascertainment or clinical practice over time.

This study has several limitations.

First, the case–control design may overestimate associations and is inherently limited in providing absolute risk estimates. Additionally, the width of the confidence intervals reflects the modest sample size, and the statistical precision was reduced when multiple covariates were included. Moreover, the strength of the association observed here cannot be directly compared with classical risk factors for recurrent SGA reported in large population‐based cohorts, where effect estimates tend to be more stable and generalizable.

Second, due to the setting of our study (low risk hospital) and the inclusion of newborn > 36 WP, some maternal factors known to be related to obstetrical outcome (maternal age, smoking) may not have the same weight, resulting in overestimating our OR.

Third, although our matched case–control design is appropriate for identifying associations, it does not allow for estimation of absolute risk or incidence. The relatively small sample size limited the power of secondary analyses. As a result, our results should be interpreted as exploratory and hypothesis‐generating rather than conclusive.

Fourth, some variables could influence effect estimates but were not fully captured such as genetic or metabolic disease, breastfeeding duration, and nausea and vomiting in pregnancy.

Additionally, the nested case–control design does not allow estimation of absolute risk of subsequent SGA, which would be of greater direct clinical utility.

## 5 CONCLUSION

In this matched case–control study, we found that infants born with low‐normal BW (≤−0.5 SD) increased the risk of SGA birth in a subsequent pregnancy. These findings highlight the potential clinical significance of subtle intrauterine growth restriction that remains undetected using conventional BW thresholds.

## AUTHOR CONTRIBUTIONS

**Beddock Richard**: Supervision. **Boujenah Jeremy**: Conceptualization; investigation; writing—original draft; methodology; software. **Patrick Rozenberg**: Methodology; validation; writing—review and editing; supervision.

## CONFLICT OF INTEREST STATEMENT

The authors declare no conflicts of interest.

## FUNDING INFORMATION

The authors received no specific funding for this work.

## ETHICS STATEMENT

The National Committee for Data Protection (Commission Nationale de l'Informatique et des Libertés, registration number 100125) approved the study, which was conducted in accordance with French legislation. Ethics committee approval was not required because the study used an anonymized database and did not influence patient care.

## Supporting information



Supporting Information

Supporting Information

Supporting Information

Supporting Information

## Data Availability

The data that support the findings of this study are available from the corresponding author upon reasonable request.
