# Supplementary material for: Low‐normal birthweight in the first pregnancy as a risk factor for small for gestational age in the second pregnancy: A matched case–control study
Source: Pregnancy (Hoboken). 2026 Jun 15;2(4):e70335. doi: 10.1002/pmf2.70335 (PMC13344237; doi:10.1002/pmf2.70335)
Supplement: Supplementary file 1 [file PMF2-2-e70335-s001.docx]

**Supplementary Table 1: Effect Modification Analysis**

| **Effect Modifier** | **Stratum** | **Crude OR (95% CI)** | **Mantel-Haenszel OR (95% CI)** | **Woolf's Test (p-value)** | **Adjusted OR performed** |
| --- | --- | --- | --- | --- | --- |
| **Overall (unstratified)** | — | 5.88 (3.68–9.40) | — | — | — |
| **ART (2nd pregnancy)** | Yes | 5.43 (3.05–9.07) | 5.85 (3.66–9.37) | 0.20 | No |
|  | No | 27.0 (1.87–1310) |  |  |  |
| **Maternal blood group** | O | 5.88 (2.65–13.12) | 5.87 (3.67–9.39) | 0.99 | No |
|  | Non-O | 5.86 (3.05–11.31) |  |  |  |
| **Family history of hypertensive disorder** | No | 6.48 (3.42–11.41) | 6.05 (3.76–9.73) | 0.57 | No |
|  | Yes | 4.62 (1.41–15.49) |  |  |  |
| **Paternal age** | < 40 years | 6.93 (4.09–11.77) | 5.73 (3.69–9.14) | 0.01 | **Yes** |
|  | ≥ 40 years | 1.16 (0.18–6.81) |  |  |  |
| **Paternal height** | < 170 cm | 5.80 (3.39–9.95) | 5.97 (3.72–9.57) | 0.77 | No |
|  | ≥ 170 cm | 7.15 (1.64–33.20) |  |  |  |
| **Interpregnancy interval** | Reference (12–48 months) | 4.43 (2.56–7.66) | 5.64 (3.54–8.89) | 0.05 | No |
|  | Short (≤ 12 months) | 37.92 (5.79–386.6) |  |  |  |
|  | Long (≥ 48 months) | 8.89 (1.07–83.83) |  |  |  |

*Effect modification was assessed using stratified bivariate analyses and Mantel-Haenszel pooled estimates. A variable was considered a potential effect modifier if stratum-specific odds ratios differed substantially and if Woolf's test suggested heterogeneity (p < 0.10). An adjusted analysis was performed only when significant effect modification was identified. ART: Assisted Reproductive Technology; CI: Confidence Interval; IPI: Interpregnancy Interval; OR: Odds Ratio; OR_MH: Mantel-Haenszel pooled Odds Ratio.*

**Supplementary Table 2: Sensitivity Analysis According to Alternative Birthweight Reference Curves**

| **Growth Reference Curve** | **Outcome (SGA cases)** | **Exposure Category (reference: Z-score > −0.5 SD)** | **Adjusted OR (95% CI)** |
| --- | --- | --- | --- |
| **AUDIPOG** | SGA (n = 125) | > −0.5 SD (ref, n = 191) | Reference |
|  |  | −0.5 to −1.0 SD (n = 138) | 5.11 (2.51–10.41) |
|  |  | ≤ −1.0 SD (n = 46) | 8.52 (3.40–21.30) |
| **FENTON** | SGA (n = 119) | > −0.5 SD (ref, n = 224) | Reference |
|  |  | −0.5 to −1.0 SD (n = 98) | 3.52 (1.74–7.11) |
|  |  | ≤ −1.0 SD (n = 53) | 6.53 (2.73–15.62) |
| **INTERGROWTH-21st** | SGA (n = 108) | > −0.5 SD (ref, n = 290) | Reference |
|  |  | −0.5 to −1.0 SD (n = 66) | 7.01 (3.26–15.10) |
|  |  | ≤ −1.0 SD (n = 19) | 17.52 (4.40–69.79) |

*All models adjusted for maternal age, maternal height, pre-pregnancy weight, smoking during pregnancy, ethnicity, time to conceive, and socioeconomic status. Children reclassified as non-SGA under alternative references were excluded from the respective sensitivity analyses. CI: Confidence Interval; OR: Odds Ratio; SD: Standard Deviation; SGA: Small for Gestational Age.*

**Supplementary Table 3: Robustness of the Association Between Low First Birthweight and Subsequent SGA — Comparison of Statistical Models**

| **Model** | **Research Question** | **Exposure Level (ref. > −0.5 SD)** | **Adjusted OR (95% CI)** | **Key Statistic & Interpretation** |
| --- | --- | --- | --- | --- |
| **Standard logistic regression (no clustering)** | Is low first birthweight associated with subsequent SGA after adjustment for confounders? | −0.5 to −1.0 SD | 5.11 (2.51–10.41) | Pseudo R² = 0.43 Significant association after adjustment.  Does not account for shared latent factors. |
|  |  | < −1.0 SD | 8.52 (3.40–21.30) |  |
| **Cluster-robust logistic regression (patient-level clustering)** | Does the association persist after accounting for within-woman correlation? | −0.5 to −1.0 SD | 4.36 (2.54–7.48) | Pseudo R² = 0.17 Association robust to within-woman correlation. Increases precision. |
|  |  | < −1.0 SD | 8.33 (3.93–17.67) |  |
| **Generalized Structural Equation Model (GSEM)** | Does the association suggest a shared latent factor across pregnancies? | −0.5 to −1.0 SD | 5.46 (2.57–11.60) | ρ = 0.33 (95% CI: 0.23–0.41)  Statistical evidence for a shared latent factor (uteroplacental dysfunction). |
|  |  | < −1.0 SD | 8.00 (3.71–17.20) |  |
| **Mixed-effects logistic regression (random intercept per woman)** | Is the association driven by between-woman heterogeneity? | −0.5 to −1.0 SD | 5.11 (2.51–10.41) | ICC ≈ 0 Between-woman variability is negligible, supporting a fixed underlying mechanism. |
|  |  | < −1.0 SD | 8.52 (3.40–21.30) |  |

*All models use the same covariates: maternal age, maternal height, pre-pregnancy weight, smoking during pregnancy, ethnicity, time to conceive, and socioeconomic status. GSEM: Generalized Structural Equation Model; ICC: Intraclass Correlation Coefficient; OR: Odds Ratio; ρ: correlation of unmeasured residuals; SD: Standard Deviation; SGA: Small for Gestational Age.*
