# Supplementary material for: Low‐normal birthweight in the first pregnancy as a risk factor for small for gestational age in the second pregnancy: A matched case–control study
Source: Pregnancy (Hoboken). 2026 Jun 15;2(4):e70335. doi: 10.1002/pmf2.70335 (PMC13344237; doi:10.1002/pmf2.70335)
Supplement: Supplementary file 2 [file PMF2-2-e70335-s003.docx]

**Supplementary Figure 2** *— Interaction with body mass index*

Marginal effect analysis was conducted on interpregnancy BMI change to illustrate the variation of the exposure effect across BMI levels and to detect potential non-linear patterns. Predictive margins represent the estimated probability of SGA at the second pregnancy for each exposure group across standardized BMI values, with 95% confidence intervals.

**Figure legend.** Predictive margins of SGA probability at the second pregnancy by exposure status and standardized interpregnancy BMI change. **E = 0**: first child birthweight Z-score ≥ −0.5 SD (non-exposed). **E = 1**: first child birthweight Z-score < −0.5 SD (exposed). Error bars and shaded areas represent 95% confidence intervals.

BMI values are standardized (mean = 0, SD = 1). The impact of prior low-normal birthweight (E = 1) **declines with increasing maternal BMI** (p < 0.05), with the strongest effect observed at low BMI values, consistent with a modifying role of nutritional status on uteroplacental risk.
