# Supplementary material for: Low‐normal birthweight in the first pregnancy as a risk factor for small for gestational age in the second pregnancy: A matched case–control study
Source: Pregnancy (Hoboken). 2026 Jun 15;2(4):e70335. doi: 10.1002/pmf2.70335 (PMC13344237; doi:10.1002/pmf2.70335)
Supplement: Supplementary file 3 [file PMF2-2-e70335-s004.docx]

**Supplementary Table 4** *—* Quantitative Bias Analysis

**Part A. Deterministic Quantitative Bias Analysis (QBA)**

Unmeasured or misclassified confounding factors considered**:** partner change between pregnancies, non-CMV infections, alcohol or toxic exposures, special diet.

A quantitative bias analysis (QBA) was conducted to assess the potential impact of an unmeasured confounder. Three scenarios were simulated combining varying differences in the prevalence of this hypothetical confounder between exposed and unexposed groups (ranging from 15 to 35 percentage points) and different strengths of association with the outcome (risk ratio 2 to 4).

| **Scenario** | **Risk ratio of unmeasured confounder** | **Prevalence among exposed (%)** | **Prevalence among unexposed (%)** | **Bias-adjusted OR** |
| --- | --- | --- | --- | --- |
| **Moderate** | 2 | 30 | 15 | 4.23 |
| **Severe** | 3 | 35 | 10 | 3.24 |
| **Extreme** | 4 | 40 | 5 | 2.37 |

*Even in the most extreme scenario (prevalence 40% vs. 5%, RR = 4), the bias-adjusted OR remained at 2.37, suggesting that residual confounding alone is unlikely to fully explain the observed association (OR = 4.86).*

**Part B. Probabilistic Sensitivity Analysis — Monte Carlo Simulation**

Results of 10,000 Monte Carlo simulations testing the potential impact of an unmeasured confounder. Each iteration sampled random values for the prevalence of the confounder among exposed and unexposed individuals (20–50% vs. 5–30%), and the confounder's strength of association with the outcome (risk ratio 1.5–4.0).

**Figure legend.** Distribution of bias-adjusted odds ratios across 10,000 simulated scenarios. The grey dashed line indicates the median bias-adjusted OR (≈ 3.86); the red dashed line represents the observed OR (4.86).

The median bias-adjusted odds ratio was 3.86. Even in the most extreme simulated scenarios, the association remained above 2.1. The distribution suggests that under any plausible scenario of unmeasured confounding, the association between low-normal first birthweight and subsequent SGA remains substantial.
